# Analysis of the Polymorphisms and Expression Levels of the BCL2, BAX and c-MYC Genes in Patients with Ovarian Cancer

**DOI:** 10.3390/ijms242216309

**Published:** 2023-11-14

**Authors:** Piotr Józef Olbromski, Anna Bogacz, Marta Bukowska, Adam Kamiński, Rafał Moszyński, Piotr Pawlik, Anna Szeliga, Katarzyna Kotrych, Bogusław Czerny

**Affiliations:** 1 Clinic of Operational Gynecology, Poznan University of Medical Sciences, Polna 33, 60-535 Poznan, Poland; olbromski.piotr@gmail.com (P.J.O.); pitpawlik@gmail.com (P.P.); 2 Department of Personalized Medicine and Cell Therapy, Regional Blood Center, Marcelińska 44, 60-354 Poznan, Poland; marta-bukowska4@wp.pl; 3 Department of Orthopedics and Traumatology, Independent Public Clinical Hospital No. 1, Pomeranian Medical University, UniiLubelskiej 1, 71-252 Szczecin, Poland; emluc@wp.pl; 4 Department of Gynecology, Obstetrics and Gynecological Oncology, Poznan University of Medical Sciences, Polna 33, 60-535 Poznan, Poland; rafalmoszynski@gmail.com; 5 Department of Gynecological Endocrinology, Poznan University of Medical Sciences, Polna 33, 60-535 Poznan, Poland; annamaria.szeliga@gmail.com; 6 Department of General and Dental Radiology, Pomeranian Medical University in Szczecin, al. Powstańców Wielkopolskch 72, 70-111 Szczecin, Poland; kotrych1@gmail.com; 7 Department of Pharmacology and Pharmacoeconomics, Pomeranian Medical University in Szczecin, Żołnierska 48, 71-230 Szczecin, Poland; boguslaw.czerny@iwnirz.pl

**Keywords:** ovarian cancer, gene expression, biomarkers, protein expression, single-nucleotide polymorphism, individualized therapy

## Abstract

Ovarian cancer (OC) is one of the biggest problems in gynecological oncology and is one of the most lethal cancers in women worldwide. Most patients with OC are diagnosed at an advanced stage; therefore, there is an urgent need to find new biomarkers for this disease. Gene expression profiling is proving to be a very effective tool for exploring new molecular markers for OC patients, although the relationship between such markers and patient survival and clinical outcomes is still elusive. Moreover, polymorphisms in genes encoding both apoptosis-associated proteins and oncoproteins may serve as key markers of cancer susceptibility. The aim of our study was to analyze the polymorphisms and expressions of the *BCL2*, *BAX* and *c-MYC* genes in a group of 198 women, including 98 with OC. The polymorphisms and mRNA expressions of the *BCL2*, *BAX* and *c-MYC* genes were analyzed using real-time PCR. The analysis of the *BAX* (rs4645878; G>A) and *c-MYC* (rs4645943; C>T) polymorphisms showed no association with ovarian cancer risk. The *BCL2* polymorphism (rs2279115; C>A) showed a significant difference in the frequency of genotypes between the studied groups (CC: 23.47% vs. 16.00%, AA: 25.51% vs. 37.00%; *p* = 0.046; OR = 1.61). Furthermore, the expression levels of the *BCL2* and *c-MYC* genes showed a decrease at the transcript level for OC patients compared to the control group (*BCL2*: 17.46% ± 3.26 vs. 100% ± 8.32; *p* < 0.05; *c-MYC*: 37.56% ± 8.16 vs. 100% ± 9.12; *p* < 0.05). No significant changes in the mRNA level were observed for the *BAX* gene (104.36% ± 9.26 vs. 100% ± 9.44; *p* > 0.05). A similar relationship was demonstrated in the case of the protein expressions of the studied genes. These findings suggest that the CC genotype and C allele of the *BCL2* polymorphism could be genetic risk factors for OC development. A gene expression analysis indicated that *BCL2* and *c-MYC* are associated with OC risk.

## 1. Introduction

Ovarian cancer (OC) is one of the most lethal cancers and the most fatal gynecologic malignancy, causing more than 100,000 deaths each year around world [1,2,3]. The frequency of OC worldwide varies. According to Gaona-Luviano et al., the highest morbidity of OC is noted in Northern Europe and the USA. Conversely, the lowest morbidity of OC is registered in Japan. Several different risk factors of OC are described in the literature, like age, reproductive health, demographic status, genetics, gynecologic status, hormonal factors and those related to lifestyle [3].

Ovarian cancer can be divided into epithelial and non-epithelial types. Epithelial ovarian cancer (EOC) is, in particular, an extremely aggressive type of tumor [1,4]. In early stages, OC can be successfully treated. Unfortunately, due to it having mostly non-specific symptoms, the disease is often detected in advanced stages with extensive spread and a poor survival ratio [2,5].

OC is mainly diagnosed at stages 3 and 4 due to its silent and insidious development and lack of early symptoms. The conventional treatment of OC involves cytoreductive surgery, which is followed by adjuvant platinum-based chemotherapy [6]. Cell therapies, which provide exciting results for hematological malignancies, have not yet been repeated in, for example, EOC. Unfortunately, the “perfect” target antigen, which would be highly and homogeneously expressed by the tumor and minimally expressed by healthy tissues, still has not been found [4]. Furthermore, immunotherapy in OC is still not as successful as in other types of cancer [7]. It is worth mentioning that, despite well-known and aggressive treatment, recurrence occurs in about 70% of patients within 2–3 years [6].

To improve OC detection at early stages, it is important to develop screening tests with high specificity (around 99.6%), high sensitivity (about 75%) and a positive predictive value of 10%. Nowadays, we use the following detection methods in OC diagnostics as “gold standards”: pelvic examination, transvaginal ultrasonography (TVUS) and the measurement of serum CA-125 levels. CA-125 is a classic tumor biomarker that is widely used in cancer therapy. At this point, it is important to point out that CA-125 is not exclusively expressed on ovarian tumor cells. Nevertheless, the level of CA-125 is useful for monitoring the recurrence of OC. As an early detection marker, the significance of CA-125 is undermined. About 20% of OCs do not express CA-125. Therefore, CA-125 is not sufficiently accurate as a detection marker. However, the combined levels of CA-125 and HE4 (a protein that is overexpressed in OC) have higher sensitivity (about 76.4%) and specificity (around 95%) than either of them alone. Additionally, VEGF (vascular endothelial growth factor) is involved in the progression of the tumor and metastasis in OC. The higher the VEGF level, the shorter the disease-free survival and overall survival. It was found that VEGF was elevated in 81% of cases of OC when CA-125 was deficient. Adding VEGF to CA-125 and HE4 increased sensitivity up to 84% in stage I [8].

It is worth mentioning that the study of predisposition to OC is as important as progress in modern diagnostics. Recently, the polymorphisms and expression levels of three genes have been studied: *BCL2*, *BAX* and *c-MYC*.

*c-MYC* plays a crucial role in biological and cellular regulations. Its role in tumorigenesis and cancer progression has been brought to attention. It appears that *c-MYC* plays a role in the proliferation and metastasis of tumors. *c-MYC* is associated with chemotherapy resistance and poorer clinical outcomes. However, targeting *c-MYC* has become an essential step in molecular therapy in human cancers. Genetic studies have shown that suppressing the activation of *c-MYC* can cause tumor regression by inhibiting proliferation and inducing apoptosis [9].

*BCL-2* is known for its role in maintaining the internal environment of airway epithelial cells. Additionally, *BCL2* is associated with autophagy through the encoding of a mitochondrial membrane protein that blocks the apoptosis of lymphocytes [10]. The overexpression of *BCL-2* and *BAX* plays an important role in hematopoietic cancers [11]. The most common *BCL-2* polymorphism, BCL-2 −938C>A in the promoter region of the gene, has been associated with predisposition to breast cancer. Conversely, *BAX* −248G>A is a polymorphism associated with several cancers [12]. The aim of this study is to analyze the polymorphisms and expressions of the *BCL2*, *BAX* and *c-MYC* genes in patients with ovarian cancer.

## 2. Results

Sociodemographic data were collected from 98 women with ovarian cancer and 100 healthy women, and they included age, education, occupation and economic status. In Table 1, it can be observed that the highest number of women with ovarian cancer aged above 40 years was 89 patients (90.12%). Similar values were observed in the control group (88.00%). The majority of the respondents were women with secondary education (36.73%), followed by women with vocational education (32.65%), and higher education was declared by 30.62% of the surveyed population. In the control group, the values were distributed similarly (secondary education—38.00%; vocational education—32.00%; and higher education—30.00%). Considering the occupation category in the group of women with ovarian cancer, the occupation of other manual worker accounted for 53.06%. The occupations of farmers and office worker or other specialist accounted for 16.33% and 12.25%, respectively. In the control group, the following values were recorded: other manual workers—42.00%; office workers or other specialists—21.00%; farmers—14.00%; teachers/educators—10.00%; health professionals—7.00%; and unemployed—6.00%. A good economic status was reported by 58 women with ovarian cancer (59.18%), which was similar to the controls (56.00%). The sociodemographic characteristics of the patients are described in Table 1.

A comparison of selected clinical and biochemical parameters between the women with ovarian cancer and the control group is presented in Table 2. Blood counts, such as leukocytes, erythrocytes, platelets and hemoglobin, showed significant differences between the women with ovarian cancer and the control group. In parameters such as D-dimer (3349.653 ng/mL ± 2024.42 vs. 790.69 ng/mL ± 424.54, *p* < 0.001), fibrinogen (7.65 g/L ± 5.34 vs. 2.98 g/L ± 0.78, *p* < 0.001), CA-125 (778.51 U/mL ± 444.25 vs. 128.29 U/mL ± 100.43, *p* < 0.001) and HE4 (1709.42 pmol/L ± 1208.42 vs. 84.47 pmol/L ± 31.30, *p* = 0.008), statistical differences between the study groups were demonstrated.

The genotype and allele frequencies of the selected polymorphisms and their associations with ovarian cancer risk are summarized in Table 3. The genotype distributions of the selected polymorphisms are consistent with HWE. The chi square (χ^2^) test for the genotype distributions between the tumor patients and cancer-free controls were *p* = 0.046 for rs2279115, C>A; *p* = 0.401 for rs4645878, G>A; and *p* = 0.331 for rs4645943, C>T.

The analysis of *BAX* (rs4645878, G>A) and *c-MYC* (rs4645943, C>T) polymorphisms showed no association with ovarian cancer risk (Table 3). There were no differences in the genotype distribution between the patients with ovarian cancer and the healthy women. The analysis of the *BCL2* polymorphism (rs2279115, C>A) showed a significant difference in the frequency of genotypes between the studied groups (CC: 23.47% vs. 16.00%, AA: 25.51% vs. 37.00%; *p* = 0.046, OR = 1.61). Based on the results obtained for the *BCL2* polymorphism (rs2279115), it can be concluded that patients with the CC genotype and the C allele (48.98% vs. 39.50%, *p* = 0.039) show an increased risk of ovarian cancer.

**Figure 1:**
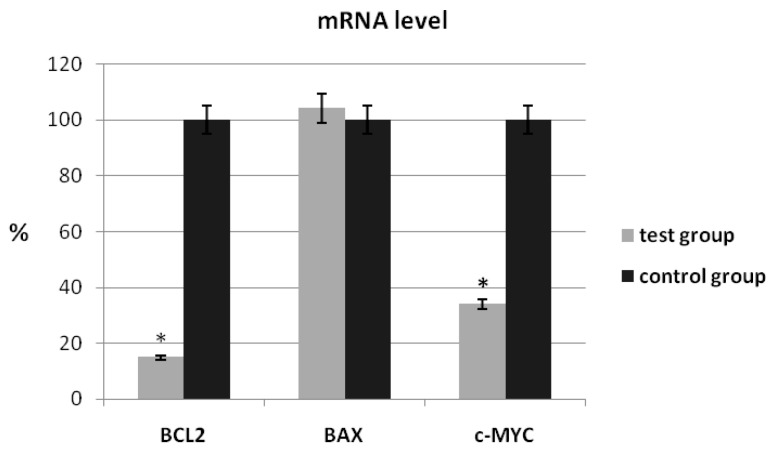
Analysis of mRNA expression of selected genes in patients with ovarian cancer compared to the control group. The control group was defined as 100%. Data are presented as mean% ± SD, * *p* < 0.05 as compared with the control group.

In our study, we also assessed the expression levels of the *BCL2*, *BAX* and *c-MYC* genes through the real-time PCR technique. The analysis of the expression levels of the *BCL2* and *c-MYC* genes showed a decrease in the transcript level in patients with ovarian cancer compared to the control group (*BCL2*: 17.46% ± 3.26 vs. 100% ± 8.32; *p* < 0.05, *c-MYC*: 37.56% ± 8.16 vs. 100% ± 9.12; *p* < 0.05) (Figure 1). No significant changes in the mRNA level were observed for the *BAX* gene (104.36% ± 9.26 vs. 100% ± 9.44; *p* > 0.05).

The analysis of the protein level showed a statistically significant decrease in the expression of *BCL2* (1.452 ± 0.002 vs. 2.324 ± 0.002; *p* < 0.05) compared to the control group (Table 4). In the case of *c-MYC*, the decrease in the protein level was not statistically significant (0.725 ± 0.003 vs. 1.122 ± 0.003; *p* = 0.058). No change in expression level was observed for *BAX*.

## 3. Discussion

Ovarian cancer accounts for a significant number of deaths among all gynecological cancers. The disease is initially asymptomatic and is therefore usually detected in advanced stages, where complete recovery is usually impossible. Despite ongoing research, there are still no effective biomarkers with adequate diagnostic sensitivity and specificity or other effective diagnostic methods that enable screening in a timely manner. The search for diagnostic, prognostic and predictive biomarkers is currently underway. New methods of targeted therapy are also sought, aimed at blocking the tumor-specific molecular pathways responsible for its development and expansion.

Over the past few decades, mRNA evaluation has been widely used in the identification and development of new molecular biomarkers for the diagnosis and treatment of many types of cancer. It is believed that an mRNA analysis can allow for an early and more accurate prediction and prognosis of the disease and its progression, as well as allowing for the identification of patients at risk.

In our study, we analyzed the polymorphisms and expressions of the *BCL2*, *BAX* and *c-MYC* genes in patients with ovarian cancer. Sociodemographic data, including age, education, occupation and economic status, as well as selected clinical and biochemical parameters, were analyzed among the studied Caucasian population from the Greater Poland region. Our analysis of sociodemographic data did not reveal any significant differences between the women with OC and the control group. Therefore, the studied sociodemographic data did not influence the genetic differences in our population. It was observed that the largest group participating in this study comprised women over 40 years of age (OC: 90.12% vs. controls: 88.00%), with secondary education (OC: 36.73% vs. controls: 38.00%), occupation as other manual worker (OC: 53.06% vs. controls: 42.00%) and a good economic status (OC: 59.18% vs. controls: 56.00%).

The analysis of selected clinical and biochemical parameters between the women with OC and the control group showed significant differences in blood counts, such as leukocytes, erythrocytes, platelets and hemoglobin. We also noted statistical differences between the study groups in parameters such as D-dimer (3349.653 ng/mL ± 2024.42 vs. 790.69 ng/mL ± 424.54, *p* < 0.001), fibrinogen (7.65 g/L ± 5.34 vs. 2.98 g/L ± 0.78, *p* < 0.001), CA-125 (778.51 U/mL ± 444.25 vs. 128.29 U/mL ± 100.43, *p* < 0.001) and HE4 (1709.42 pmol/L ± 1208.42 vs. 84.47 pmol/L ± 31.30, *p* = 0.008), which indicates correlations between the tested parameters and the degree of disease advancement.

Our analysis of the expression levels of the *BCL2* and *cMYC* genes showed a decrease in the transcript level (*BCL2*: 17.46% ± 3.26 vs. 100% ± 8.32; *p* < 0.05, *cMYC*: 37.56% ± 8.16 vs. 100% ± 9.12; *p* < 0.05) in the patients with ovarian cancer compared to the control group. No significant changes in the mRNA level was observed for the *BAX* gene. Similarly, a decrease in the protein level was observed for *c-MYC* (0.725 ± 0.003 vs. 1.122 ± 0.003; *p* = 0.058) and *BCL2* (1.452 ± 0.002 vs. 2.324 ± 0.002; *p* < 0.05). No change in the protein level was observed for BAX. This is the first study to analyze the expressions of the *c-MYC*, *BAX* and *BCL2* genes among the Polish population. As research shows, *BCL2* expression is extremely complex in ovarian cancer cells and tissues. Yuan et al., when detecting *BCL2* using 12 cell lines derived from human ovarian cancer cells, showed that the level of *BCL2* expression was negatively correlated with cisplatin sensitivity [13]. The BCL2 protein expression was low in ovarian-cancer-resistant cell lines, such as SKOV-3, 59M and OVCAR-3. In contrast, a high expression of *BCL2* was observed in sensitive ovarian cancer cell lines (41M and CH1) [13]. In addition, it was noted that *BCL2* expression also differed in various cell lines. *BCL2* expression was shown to be almost undetectable in the cisplatin-resistant A2780 cell line [14]. However, another study by Liang and Zhao showed an increased expression of the BCL2 protein in ovarian cancer tissues in the case of lymphoid metastases and post-operative recurrence tissue. Increased BCL2 protein expression in ovarian cancer tissues was also associated with tumor stage [15]. In our study, *BCL2* expression was lower in tumor tissue similarly to ovarian-cancer-resistant cell lines. However, the results of various studies indicate an ambiguous level of *BCL2* expression associated with ovarian cancer, which is a more complex molecular aspect in the pathogenesis of this cancer.

However, *BCL2* may function as an oncogene or as a tumor suppressor gene in various types of cancer. For example, higher levels of *BCL2* expression are associated with poor survival of patients with chronic lymphocytic leukemia (CLL) but improved survival of patients with breast and colon cancer [16,17,18]. Many studies have clearly shown that an increase in *BCL2* expression is associated with improved treatment in breast cancer [17,19,20]. It has also been proven that *BCL2* expression is an independent indicator of favorable prognosis for all types of breast cancer in the early stages [17,19].

Moreover, our analysis of the *BCL2* polymorphism (rs2279115, C>A) showed a significant difference in the frequency of genotypes between the studied groups (CC: 23.47% vs. 16.00%, OR = 1.61; AA: 25.51% vs. 37.00%, OR = 1.74; *p* = 0.046). The distribution of genotype and allele frequencies for the rs2279115 polymorphism is consistent with HWE. Based on the results obtained for the *BCL2* polymorphism (rs2279115), it can be concluded that the patients with the CC genotype and the C allele (48.98% vs. 39.50%, *p* = 0.039) showed an increased risk of ovarian cancer.

As previously shown, the C allele, compared to the A allele, significantly increases the inhibition of *BCL2* promoter activity and the binding of nuclear proteins [21]. Our research shows a similar relationship. We observed decreased *BCL2* expression, which may be caused by an increased frequency of the CC genotype (23.47% vs. 16.00%, *p* = 0.046) and the C allele (48.98% vs. 39.50%, *p* = 0.039) of the rs2279115 (−938C>A)polymorphism, in the study group compared to the control group among Polish women. Consistent with these results, the BCL2 protein expression in the B cells of CLL patients with the AA genotype was significantly higher than in those with the CC genotype [21]. This relationship was also demonstrated in relation to lymph-node-negative breast cancer, where a higher BCL2 expression was associated with the A allele (*p* = 0.044), and a Kaplan–Meier survival analysis revealed a significant association between the AA genotype and improved survival (*p* = 0.030) [22]. This association was also demonstrated in oropharyngeal squamous cell carcinoma, where the rs2279115 polymorphism was significantly associated with *BCL2* expression (*p* = 0.008) and overall survival (*p* = 0.025) [23]. This tendency has also been observed in kidney cancer [24]. We found a similar relationship in our study regarding *BCL2* expression and polymorphic variants affecting *BCL2* expression between patients with ovarian cancer and the control group.

Another study by Ozoran et al. found no association between the −938C>A polymorphism of the *BCL2* gene and breast cancer [12]. Similar conclusions were obtained by Searle et al. [25]. Zhang et al. conducted a study on the relationship between the −938C>A polymorphism of the *BCL2* gene and breast cancer. They found that patients with the AA genotype had a 2.37 times higher risk of developing breast cancer than those with the AC and CC genotypes [26]. It is suggested that the relationship between the risk of cancer development and the prognosis of patient survival may result from the expression of this gene and the complexity of the molecular background of cancer.

The *BAX* gene is involved in tumor suppression due to its role in promoting programmed cell death. *BAX* has been extensively studied in many types of cancer, including pancreatic cancer [27], colon cancer [28], ovarian cancer [29], lung cancer [30] and breast cancer [31]. In our study, we also assessed *BAX* expression levels in ovarian cancer. According to a study on ovarian cancer cells, there was no significant increase in the drug susceptibility of tumor cells with *BAX* overexpression [14]. A tissue-level analysis of the *BAX* expression in 45 patients with epithelial ovarian cancer showed that patients with higher *BAX* levels were completely sensitive to chemotherapy, while patients with lower *BAX* levels were resistant to treatment [32]. The results obtained in our study showed no changes in the expression of *BAX.* Therefore, it can be concluded that these studies show an ambiguous *BAX* expression level in patients with ovarian cancer, indicating a variable level of *BAX* expression in this cancer.

In addition, some polymorphisms, such as *BAX* −248G>A (rs4645878) and *BCL2* −938C>A (rs2279115), have been determined to exhibit changes in gene expression contributing to diseases [33]. It has been shown that deletions of the *BAX* gene may be associated with the occurrence of lymphoid hyperplasia. Hence, this gene is considered to be an important suppressor of hematopoietic malignancies [34]. In the case of polycythemia vera or essential thrombocythemia, there was no significant difference in the frequency of genotypes for the *BAX* −248G>A (rs4645878) polymorphism between the patients and the control group [33]. Our analysis of BAX (rs4645878, G>A) and c-MYC (rs13281615, A>G) polymorphisms also showed no association with ovarian cancer risk. There were no differences in the genotype distribution between the patients with ovarian cancer and the healthy women (for *BAX* GG: 75.51% vs. 73.00%, OR = 1.14; GA: 23.47% vs. 25.00, OR = 1.09; AA: 1.02 vs. 2.00, OR = 1.10; *p* = 0.401; for *c-MYC* AA: 33.67% vs. 38.00%, OR = 0.82; AG: 42.86% vs. 42.00%, OR = 0.96; GG: 23.47% vs. 20.00%, OR = 0.81; *p* = 0.331). Moreover, the distribution of genotype and allele frequencies for the rs4645878 and rs13281615 polymorphisms is consistent with HWE. The analysis of allele frequencies for the rs4645878 and rs13281615 polymorphisms also showed no relationship with the risk of ovarian cancer (for rs4645878 *BAX* G: 87.25% vs. 85.50, A: 12.75% vs. 14.50%; OR = 1.16; *p* = 0.062; for rs13281615 *c-MYC* A: 55.10% vs. 59.00%, G: 44.90% vs. 41.00%; OR = 0.76; *p* = 0.642).

Our results are consistent with those of studies on solid tumors, such as the study reported by Alam et al. [35]. Although *BAX* rs4645878 does not appear to play a role in carcinogenesis, it has been suggested that it may be associated with poor prognosis in some cancers [36].

In a study by Ozoran et al., women with the AA genotype for the *BAX* −248G>A polymorphism had a 5 times greater risk of developing breast cancer. In addition, metastatic status and tumor size were associated with this genotype [12]. Similar results were obtained in a study by Kholoussi et al. [37]. They showed that the presence of the GA and AA variants of the *BAX* −248G>A polymorphism is associated with the risk of cancer development. The relationship between the −248G>A polymorphism of the *BAX* gene and clinical parameters in different types of cancer has previously been analyzed with various results. Wang et al. studied the effect of the −248G>A polymorphism of the *BAX* gene on survival in gastric cancer patients receiving postoperative chemotherapy. They showed that one of the variants, the −248G>A polymorphism, of the *BAX* gene was associated with the risk of disease recurrence and had a weak impact on survival [38]. Gu et al. studied the association of the *BAX* −248G>A polymorphism with hematologic toxicity in patients with advanced-stage small-cell lung cancer receiving platinum-based chemotherapy. They showed that the −248G>A *BAX* polymorphism did not affect survival [39].

In contrast, Saxena et al. showed that the BAX −248G>A polymorphism was associated with protein expression [40]. Compared to the A allele, Starczyński et al. reported that G alleles are associated with higher levels of mRNA and protein [41]. However, Yu et al. believed that this polymorphism was associated with lower transcriptional activity [42]. Skogsberg et al. showed that the polymorphism of the *BAX* gene is not related to its expression [43]. We obtained similar results in our study in a Caucasian population, showing no relationship between the rs4645878 (−248G>A)polymorphism and the expression of the *BAX* gene. However, another study showed that a low expression of the Bax protein suggests a poor prognosis [44].

Some researchers found that mutations in the *BAX* gene led to a reduced expression of its protein, which was closely related to drug resistance. They believed that the *BAX* gene polymorphism was associated with a poor prognosis [44]. However, Skogsberg et al. found that the *BAX* polymorphism was not associated with the prognosis of cancer patients [43]. Similar conclusions were obtained in our study.

*C-MYC*, encoding the c-Myc protein, is an important oncogene involved in many stages of tumorigenesis, such as proliferation, survival and apoptosis. The c-Myc oncoprotein is overexpressed in a large number of human cancers, and its expression is associated with poor prognosis [45]. It has been shown that *c-MYC* expression can be downregulated with statins. The possibility that *c-Myc*-expressing tumors can be treated with statins has been demonstrated. As discussed above, statins reduce *c-MYC* biosynthesis. Wu et al. showed that the inactivation of the c-Myc protein promotes tumor senescence [46]. In our study, we showed a decrease in the *cMYC* expression level (37.56% ± 8.16 vs. 100% ± 9.12; *p* < 0.05) and protein level (0.725 ± 0.003 ng/mL vs. 1.122 ± 0.003 ng/mL; *p* = 0.058) in patients with ovarian cancer compared to the control group from Poland. This is the first study in the Polish population to take into account the analysis of the *c-MYC* gene expression level and protein level in ovarian cancer.

The rs4645943 C>T polymorphism is located in the 5′ UTR of the *MYC* gene, a key region for regulating its expression. The rs4645943 C>T polymorphism has been reported to be associated with prostate cancer risk [47]. However, no study has shown an association between this polymorphism and the risk of developing ovarian cancer. Liu et al. showed that both *MYC* gene-related polymorphisms (rs4645943, C>T and rs2070583, A>G) were not associated with Wilms tumor risk in the Chinese population [48]. In our population, the analysis of the *c-MYC* polymorphism (rs4645943, C>T) also did not show any association with the risk of ovarian cancer.

A study by Pan et al. showed that the polymorphism of the *MYC* gene (rs4645943 and rs2070583) may have a weak effect on the risk of neuroblastoma, which requires further verification [49]. Another study showed that the *c-MYC* rs4645943 and rs2070583 polymorphisms were not associated with the risk of hepatocytoma [50].

Clinical data on the levels of expression and polymorphisms of the studied genes in the development of ovarian cancer in this study were scarce. This may be due to the complexity of the neoplastic process and the variability in the expressions of genes involved in its development or progression.

## 4. Materials and Methods

### 4.1. Patients

A total of 198 patients from a Caucasian population were recruited in the Clinical Hospital of the Poznan University of Medical Sciences (Poland). Ovarian cancer was diagnosed in 98 women (mean age: 58 years) based on histological tests. According to FIGO (International Federation of Gynaecology and Obstetrics), the majority of women in the study group (80%) had stage III ovarian cancer with poor differentiation. The control group included 100 women (mean age: 64 years) operated for uterine fibroids or prolapse of the reproductive organ after menopause without any history of cancer. Tissue samples from 100 normal ovaries and 98 ovarian carcinoma tumors were used to evaluate RNA and protein expressions. Blood samples were used to analyze the *BCL2*, *BAX* and *c-MYC* polymorphisms. This study was approved by the Bioethics Committee of Poznan University of Medical Sciences, Poland (no. 77/19). All patients were informed about the purpose of the study and provided written informed consent. When analyzing the demographic status, it was found that patients with ovarian cancer and women from the control group came from the Greater Poland region. Sociodemographic data were collected from them, including age, education, occupation and economic status, and they are presented in the Section 2.

### 4.2. Polymorphism Analysis of BCL2, BAX and c-MYC Genes

Genomic DNA were extracted from whole blood using a commercial NucleoSpin Blood Kit (Macherey-Nagel, Dueren, Germany) according to the protocol. The analysis of *BCL2* (rs2279115, −938C>A), *BAX* (rs4645878, −248G>A) and *c-MYC* (rs4645943, C>T) polymorphisms was performed using a LightCycler FastStart DNA MasterHybProbe (Roche Diagnostics, Filderstadt, Germany) and LightCycler^®^480 instrument (Roche Diagnostics, Germany). The determination of the polymorphic variants of the *BCL2*, *BAX* and *c-MYC* genes was carried out using LightSNIP kits containing specific primers with hybridizing probes for the tested polymorphism according to the manufacturer’s protocol.

### 4.3. Expression Analysis of BCL2, BAX and c-MYC Genes

Total RNA isolation was performed using TriPure Isolation Reagent (Roche Diagnostics, Mannheim, Germany), according to the manufacturer’s protocol. The quantity and quality of the RNA were assessed using a NanoDrop spectrophotometer (Thermo Fisher Scientific, Waltham, MA, USA). Reverse transcription was performed using a Transcriptor First Strand Synthesis Kit (Roche Diagnostics, Mannheim, Germany). The mRNA expressions of the *BCL2*, *BAX* and *c-MYC* genes were analyzed using real-time quantitative PCR. A PCR reaction and a melting analysis of the products were performed using a LightCycler480 Instrument (Roche, Mannheim, Germany) and a LightCycler480 Probes Master kit (Roche, Mannheim, Germany). Housekeeping genes, such as *GAPDH* and *β-ACTIN*, were used for the normalization of quantitative RT-PCR. The sequences of the primers that were synthesized by Genomed (Warsaw, Poland) are shown in Table 5. The data from the PCR reaction were assessed using LightCycler480 software version 1.5.

### 4.4. ELISA

A Human Bcl-2 ELISA Kit (sensitivity: 38.08 pg/mL; Abcam, Cambridge, UK), Human BAX ELISA Kit (sensitivity: 2.2 pg/mL; Abcam, Cambridge, UK) and Human c-Myc ELISA Kit (sensitivity: 0.62 ng/mL; Abcam, Cambridge, UK) were employed to evaluate the concentrations of BCL2, BAX and c-MYC from tissue homogenates according to the manufacturers’ protocols. The absorbance was measured using a microplate reader (Infinite 200, TECAN, Männedorf, Switzerland).

### 4.5. Statistical Analysis

A statistical analysis was performed with the SPSS 17.0 PL program using a one-way analysis of variance (ANOVA). Data are expressed as means ± SD. The chi square (χ^2^) test was utilized in the evaluation of the genotypes and alleles, and a test of the deviation of the genotype distribution was conducted using the Hardy–Weinberg equilibrium (HWE). The potential relationships between the *BCL2* (rs2279115), *BAX* (rs4645878) and *c-MYC* (rs4645943) genotypes and ovarian cancer cases were assessed by estimating odds ratios (ORs). Values of *p* < 0.05 were considered to indicate statistically significant differences.

## 5. Conclusions

In summary, the high rate of chemo-resistance among ovarian cancers makes this type of cancer considered the deadliest among the cancers that occur in women. The process of apoptosis plays an important role in carcinogenesis and embryogenesis, and it is under the control of the BCL-2 family proteins, the expression of which in cancer cells causes a worse prognosis. Understanding the molecular pathways that control the apoptosis of cancer cells creates new treatment options and enables the development of new methods of cancer therapy. The control of proteins from the BCL-2 family and the c-myc oncoprotein may be an invaluable research tool in modern oncology.

Our study, which is the first to study three different gene polymorphisms in the Polish population, shows that the *BCL2* polymorphism could be a genetic risk factor for OC development. A gene expression analysis indicated that *BCL2* and *c-MYC* are associated with OC risk.

Some limitations of this study should be mentioned. First, more patients are needed to further validate our results. Second, due to the lack of detailed information on the patients, this study did not analyze the associations between *BAX*, *BCL2* and *c-MYC* polymorphisms and gene expression and clinical features, such as tumor size and sensitivity to treatment.

## Figures and Tables

**Table 1:** Sociodemographic characteristics of the study population.

Parameters	Patients (N = 98)	Controls (N = 100)
**Age (years)**	**N (%)**	**N (%)**
<40	9 (9.18%)	12 (12.00%)
>40	89 (90.12%)	88 (88.00%)
**Education**	**N (%)**	**N (%)**
Primary	0 (0.00%)	0 (0.00%)
Lower secondary	0 (0.00%)	0 (0.00%)
Vocational	32 (32.65%)	32 (32.00%)
Secondary	36 (36.73%)	38 (38.00%)
Higher	30 (30.62%)	30 (30.00%)
**Occupation**	**N (%)**	**N (%)**
Farmers	16 (16.33%)	14 (14.00%)
Other manual workers	52 (53.06%)	42 (42.00%)
Office workers or other specialists	12 (12.25%)	21 (21.00%)
Health professionals	6 (6.12%)	7 (7.00%)
Teachers/educators	8 (8.16%)	10 (10.00%)
Unemployed	4 (4.08)	6 (6.00%)
**Reported economic status**	**N (%)**	**N (%)**
Bad	0 (0.00%)	0 (0.00%)
Average	32 (32.65%)	38 (38.00%)
Good	58 (59.18%)	56 (56.00%)
Very good	8 (8.17%)	6 (6.00%)

**Table 2:** Comparison of selected clinical and biochemical parameters between women with ovarian cancer and control group.

Parameter	Group	Mean ± SD	Median	95% CI	*p*
Leukocytes 10^9^/L	OC	8.44 ± 4.48	7.62	7.63–8.99	0.006
	Control	6.98 ± 3.61	6.87	6.51–7.85	
Erythrocytes 10^12^/L	OC	4.58 ± 0.68	4.42	4.36–4.50	0.150
	Control	4.39 ± 0.53	4.41	4.28–4.49	
Platelets 10^9^/L	OC	330.87 ± 155.42	288.00	295.75–370.94	0.016
	Control	266.48 ± 64.87	262.50	253.47–279.48	
Hemoglobin g/dL	OC	7.56 ± 1.01	7.54	7.36–7.68	0.036
	Control	7.61 ± 0.98	7.84	7.42–7.92	
Hematocrit	OC	0.38 ± 0.37	0.37	0.36–0.38	0.061
	Control	0.47 ± 0.88	0.39	0.32–0.65	
Glucose mg/dL	OC	98.14 ± 18.48	94.00	93.46–99.88	<0.001
	Control	88.91 ± 15.96	85.86	72.32–91.91	
Sodium mmol/L	OC	139.55 ± 2.93	138.98	138.98–140.11	0.037
	Control	139.48 ± 2.65	139.20	138.85–139.96	
Potassium mmol/L	OC	4.36 ± 0.42	4.36	4.30–4.50	0.419
	Control	4.28 ± 0.35	4.25	4.21–4.34	
Creatinine mg/dL	OC	0.85 ± 0.49	0.74	0.74–0.95	0.644
	Control	0.83 ± 0.26	0.77	0.72–0.94	
eGFR mL/min/1.73 m^2^	OC	85.63 ± 32.53	86.21	77.42–94.54	0.292
	Control	95.97 ± 24.65	96.97	82.96–107.34	
Total protein g/dL	OC	6.98 ± 0.84	6.98	6.78–7.23	0.321
	Control	7.09 ± 0.42	7.15	6.98–7.38	
Uric acid mg/dL	OC	5.28 ± 1.78	4.86	4.69–5.59	0.728
	Control	5.24 ± 1.56	5.14	4.37–5.98	
Urea mg/dL	OC	32.30 ± 18.99	27.70	27.22–36.65	0.286
	Control	33.42 ± 10.48	31.00	28.09–37.87	
D-dimer ng/mL	OC	3349.653 ± 2024.42	1931.00	2559.53–4299.54	<0.001
	Control	790.69 ± 424.54	466.50	398.20–1282.28	
Fibrinogen g/L	OC	7.65 ± 5.34	4.62	0.27–13.94	<0.001
	Control	2.98 ± 0.78	2.99	2.81–3.22	
INR	OC	1.18 ± 0.24	1.13	1.13–1.24	0.075
	Control	1.19 ± 0.07	1.12	1.09–1.14	
PTT	OC	12.99 ± 2.62	12.50	12.46–13.57	0.068
	Control	12.28 ± 0.67	12.22	11.84–12.37	
APTT	OC	30.10 ± 3.72	30.20	29.27–30.94	0.965
	Control	30.41 ± 3.22	30.55	28.84–31.98	
Systolic pressure mmHg	OC	125.27 ± 13.27	124.00	121.39–126.76	0.175
	Control	121.81 ± 14.88	120.00	118.81–124.82	
Diastolic pressure mmHg	OC	78.99 ± 14.69	80.00	76.98–82.89	0.741
	Control	79.32 ± 8.45	80.00	77.62–81.01	
CA-125 U/mL	OC	778.51 ± 444.25	295.00	505.27–1055.74	<0.001
	Control	128.29 ± 100.43	20.81	8.49–266.64	
HE4 pmol/L	OC	1709.42 ± 1208.42	364.45	128.20–3987.76	0.008
	Control	84.47 ± 31.30	74.26	8.16–141.83	

INR—international normalized ratio, PTT—prothrombin time, APTT—activated partial thromboplastin time, OC—women with ovarian cancer, eGFR—glomerular filtration rate.

**Table 3:** Genotype and allele frequencies for polymorphisms of the *BCL2*, *BAX* and *c-MYC* genes in patients with ovarian cancer and healthy women.

	Women with Ovarian Cancer		Healthy Group		*p ^a^*	OR
	Observed Values *n* (%)	Expected Values (%)	Observed Values *n* (%)	Expected Values (%)		
***BCL2* rs2279115 C>A**						
CC	23 (23.47%)	23.99	16 (16.00%)	15.60	0.046	1.61
CA	50 (51.02%)	49.98	47 (47.00%)	47.80		1.17
AA	25 (25.51%)	26.03	37 (37.00%)	36.60		1.74
Total	98 (100%)	100.0	100 (100%)	100.0		
**Allele**						
C	96 (48.98%)	-	79 (39.50%)	-	0.039	1.24
A	100 (51.02%)	-	121 (60.50%)	-		1.24
Total	196 (100.0%)	-	200 (100.0%)	-		
***BAX* rs4645878 G>A**						
GG	74 (75.51%)	76.13	73 (73.00%)	73.10	0.401	1.14
GA	23 (23.47%)	22.24	25 (25.00%)	24.80		1.09
AA	1 (1.02%)	1.63	2 (2.00%)	2.10		1.10
Total	98 (100%)	100.0	100 (100%)	100.0		
**Allele**						
G	171 (87.25%)	-	171 (85.50%)	-	0.062	1.16
A	25 (12.75%)	-	29 (14.50%)	-		1.16
Total	196 (100.0%)	-	200 (100.0%)	-		
***c-MYC* rs4645943 C>T**						
AA	33 (33.67%)	30.36	38 (38.00%)	34.81	0.331	0.82
AG	42 (42.86%)	49.48	42 (42.00%)	48.38		0.96
GG	23 (23.47%)	20.16	20 (20.00%)	16.81		0.81
Total	98 (100%)	100.0	100 (100%)	100.0		
**Allele**						
A	108 (55.10%)	-	118 (59.00%)	-	0.642	0.76
G	88 (44.9%)	-	62 (41.00%)	-		0.76
Total	196 (100.0%)	-	200 (100.0%)	-		

*^a^*, χ^2^ test for genotype distributions between tumor patients and cancer-free controls; OR, odds ratio.

**Table 4:** Analysis of the protein level (ng/mL) of BCL2, BAX and c-MYC in the tissue homogenates in women with ovarian cancer in comparison to the control group.

Gene	Patients with Ovarian Cancer	Control Group	*p*-Value *
*BCL2*	1.452 ± 0.002	2.324 ± 0.002	0.046
*BAX*	0.982 ± 0.002	0.921 ± 0.001	0.314
*c-MYC*	0.725 ± 0.003	1.122 ± 0.003	0.058

Data are presented as mean% ± SD, * *p* < 0.05 as compared with the control group.

**Table 5:** Primer sequences for real-time PCR.

Gene	Forward 5′-3′	Reverse 5′-3′	Reference
*BCL2*	CTGGTGGACAACATCGCCCT	TCTTCAGAGACAGCCAGGAGAAAT	[51]
*BAX*	CAAACTGGTGCTCAAGGCCC	GGGCGTCCCAAAGTAGGAGA	[51]
*c-MYC*	ATCTGCGACGAGGAAGAGAA	ATCGCAGATGAAGCTCTGGT	[52]
*GAPDH*	GCAAATTCCATGGCACCGT	TCGCCCCACTTGATTTTGG	[51]
*β-ACTIN*	GCCAGAGCGGGAGTGGTGAA	GGCTTGGGCTCAGGGTCATT	[53]

## Data Availability

The data presented in this study are available on request from the corresponding author.
